# Association of CSF Aβ_38_ Levels With Risk of Alzheimer Disease–Related Decline

**DOI:** 10.1212/WNL.0000000000013228

**Published:** 2022-03-01

**Authors:** Nicholas Cullen, Shorena Janelidze, Sebastian Palmqvist, Erik Stomrud, Niklas Mattsson-Carlgren, Oskar Hansson, for the Alzheimer’s Disease Neuroimaging Initiative

**Affiliations:** From the Clinical Memory Research Unit (N.C., S.J., S.P., E.S., N.M.-C., O.H.), Department of Clinical Sciences Malmö, Faculty of Medicine, and Wallenberg Center for Molecular Medicine (N.M.-C.), Lund University; Memory Clinic (S.P., E.S., O.H.), Skåne University Hospital, Malmö; and Department of Neurology (N.M.-C.), Skåne University Hospital, Lund, Sweden.

## Abstract

**Background and Objective:**

Experimental studies suggest that the balance between short and long β-amyloid (Aβ) species might modulate the toxic effects of Aβ in Alzheimer disease (AD), but clinical evidence is lacking. We studied whether Aβ_38_ levels in CSF relate to risk of AD dementia and cognitive decline.

**Methods:**

CSF Aβ_38_ levels were measured in 656 individuals across 2 clinical cohorts: the Swedish BioFINDER study and the Alzheimer's Disease Neuroimaging Initiative (ADNI). Cox regression models were used to evaluate the association between baseline Aβ_38_ levels and risk of AD dementia in AD biomarker–positive individuals (AD+; determined by CSF phosphorylated tau [P-tau]/Aβ_42_ ratio) with subjective cognitive decline (SCD) or mild cognitive impairment (MCI). Linear mixed-effects models were used to evaluate the association between baseline Aβ_38_ levels and cognitive decline as measured by the Mini-Mental State Examination (MMSE) in AD+ participants with SCD, MCI, or AD dementia.

**Results:**

In the BioFINDER cohort, high Aβ_38_ levels were associated with slower decline in MMSE score (β = 0.30 points per SD, *p* = 0.001) and with lower risk of conversion to AD dementia (hazard ratio 0.83 per SD, *p* = 0.03). In the ADNI cohort, higher Aβ_38_ levels were associated with less decline in MMSE score (β = 0.27, *p* = 0.01) but not risk of conversion to AD dementia (*p* = 0.66). Aβ_38_ levels in both cohorts were significantly associated with both cognitive and clinical outcomes when further adjusted for CSF P-tau or CSF Aβ_42_ levels.

**Discussion:**

Higher CSF Aβ_38_ levels are associated with lower risk of AD-related changes in 2 independent clinical cohorts. These findings suggest that γ-secretase modulators could be effective as disease-altering therapy.

**Trial Registration Information:**

ClinicalTrials.gov Identifier: NCT03174938.

After many promising clinical trials of Alzheimer disease (AD), only recently has a treatment been shown to potentially delay cognitive decline in a phase III clinical trial.^1-3^ In any case, effects on cognition by available treatments for AD are modest at best.^4^ Currently, the most widely accepted explanation for AD pathogenesis is the amyloid cascade hypothesis, which proposes that AD is initiated primarily by accumulation of the β-amyloid (Aβ) peptide into senile plaques, sequentially followed by the accumulation of misfolded tau protein into tangles, neuronal loss, and cognitive decline, along with loss of independence in activities of daily living.^5^

An expected consequence of the amyloid cascade hypothesis is that modulating the production of Aβ levels in the brain should prevent downstream effects of this pathology and thereby slow the disease course. It is precisely this mechanism that has been the target of recent AD therapies, although recent clinical trials have demonstrated moderate effect on disease progression as measured by cognitive tests.^6-9^ Resolving the disagreement between overwhelming evidence speaking for the role of amyloid as a disease driver and the previous failure of most (but not all) antiamyloid therapies is therefore a major unanswered question in the AD research field.

One proposed explanation for this failure is that the canonical view of amyloid accumulation may be oversimplified, particularly as it relates to the 42-amino-acid–long peptide (Aβ_42_), which has been the primary focus of fluid biomarker studies. Increasing evidence suggests that the relative abundance of different Aβ isoforms, especially those shorter than Aβ_42_, may play a more decisive role in AD pathogenesis than previously thought.^10,11^ For instance, many presenilin mutations known to cause a familial form of AD do not directly result in higher Aβ_42_ levels in the brain but rather disturb the relationship between Aβ_42_ and shorter Aβ species through a loss-of-function mechanism.^12-14^ A role for shorter Aβ species in AD development could explain why targeting of amyloid aggregates expressed primarily by Aβ_42_ levels is not sufficient to halt the trajectory of AD. This is especially relevant due to renewed interest in targeting diverse mechanisms of amyloid toxicity within the pharmaceutical industry such as γ-secretase modulators (GSMs).^15,16^ Investigating the association between shorter Aβ peptides and AD-related changes is therefore important for understanding amyloid accumulation, particularly as it relates to disease-altering therapies.

In the present study, we took a clinical approach to this question by measuring Aβ_38_ levels in CSF and characterizing them with regard to risk of developing AD dementia and cognitive decline. Our analysis was performed in 2 large, independent cohorts comprising individuals spread broadly across the AD spectrum. Our primary aim was to understand whether CSF Aβ_38_ levels relate to AD-relevant clinical outcomes and thereby shed more light on the complex relationship between the amyloid protein and AD.

## Methods

### Study Design and Participants

Participants recruited for the Swedish BioFINDER study were enrolled consecutively between 2010 and 2014 (ClinicalTrials.gov, NCT03174938). Participants consisted of consecutively included patients without dementia with mild cognitive symptoms referred to participating memory clinics as previously described.^17^ The inclusion criteria were (1) referral to the memory clinic due to cognitive symptoms experienced by the patient or informant (note that these symptoms were not necessarily memory complaints but could also be executive, visuospatial, language, praxis, or psychomotor complaints), (2) between 60 and 80 years of age, (3) baseline Mini-Mental State Examination (MMSE) score between 24 and 30 points, (4) did not fulfill criteria for any dementia, and (5) fluent in Swedish. The primary exclusion criteria were (1) significant systemic illness or organ failure, (2) ongoing alcohol or substance misuse, (3) refusal of lumbar puncture or neuropsychological assessment, and (4) cognitive symptoms that could be directly explained by another condition or disease. At baseline, patients were categorized as having either subjective cognitive decline (SCD) or mild cognitive impairment (MCI) according to an extensive neuropsychological battery examining verbal, episodic memory, visuospatial ability, and attention/executive domains.^18^ Furthermore, patients with AD who fulfilled the National Institute on Aging–Alzheimer’s Association criteria for probable AD were included in the present analysis.^19^ All participants were enrolled consecutively after being referred to a memory clinic and had follow-up visits every year. All relevant ethics committees approved the BioFINDER study, and all study participants gave written informed consent.

Additional data were analyzed from participants in the Alzheimer's Disease Neuroimaging Initiative (ADNI) study, which was launched in 2003 as a public-private partnership. Participants in the ADNI study have been recruited from >50 locations across the United States and Canada. Inclusion and exclusion criteria for ADNI have been described in detail previously.^20^ Briefly, all ADNI participants were between the ages of 55 and 90 years, had completed at least 6 years of education, were fluent in Spanish or English, and had no significant neurologic disease other than AD. Regional ethics committees of all institutions approved the ADNI study, and all study participants gave written informed consent. ADNI data were downloaded^21^ on March 1, 2020. The present analysis included only AD biomarker– positive (biomarker-positive) participants, determined from an abnormal CSF tau phosphorylated at threonine 181 (P-tau)/Aβ_42_ ratio, for which cutoffs have been previously established in both cohorts.^22^ Moreover, all participants had a diagnosis of SCD, MCI, or AD and were included in the present analysis only if they had available baseline values for age, sex, education, CSF Aβ_38_, CSF Aβ_42_, CSF P-tau, and MMSE score and at least 1 follow-up visit in which MMSE score was measured.

### Predictors and Outcomes

Demographic characteristics, including age, sex, and education, were collected from all participants. Various biomarkers of amyloid processing or pathology—Aβ_38_, Aβ_40_, Aβ_42_, and amyloid precursor protein (sAPP; available in BioFINDER only)—and P-tau were measured in CSF at baseline in all participants. All biomarkers were natural log-transformed before analysis to obtain a more normal distribution of biomarker values. In BioFINDER, Aβ_38_, Aβ_40_, Aβ_42_, P-tau, and sAPP levels were measured with a standard ELISA assay (Euroimmun, Lubeck, Germany). In ADNI, Aβ_38_, Aβ_40_, and Aβ_42_ levels in CSF were measured with a 2-dimensional ultraperformance liquid chromatography tandem mass spectrometry method at the University of Pennsylvania (first made publicly available in February 2020). CSF P-tau levels in ADNI were measured with the Elecsys platform (Roche, Basel Switzerland).

The primary outcome was longitudinal change in cognition as measured by the MMSE scale. MMSE is a cognitive test that is highly relevant to cognitive changes in AD and is often used as a basis for making a clinical diagnosis or for inclusion in clinical trials.^23^ The secondary clinical outcome was the Preclinical Alzheimer's Cognitive Composite (PACC) score, which was developed specifically to identify early cognitive changes in individuals without dementia.^24^ The modified PACC score used in the current study was made up of MMSE score, delayed word recall from the Alzheimer's Disease Assessment Scale–Cognitive Subscale (weighted double to reflect the emphasis on memory tests in the original PACC), animal fluency, and Trail-Making B tests. The primary clinical outcome was development of AD dementia at any time during longitudinal follow-up. Clinical status was evaluated and recorded at each follow-up visit by a physician experienced in dementia disorders. A diagnosis of AD required abnormal amyloid accumulation as evidenced by CSF or PET levels, along with consensus evaluation of Clinical Dementia Rating and the Function Activities Questionnaire.

### Statistical Analysis

Linear mixed-effects (LME) modeling was used to assess the relationship between continuous Aβ_38_ levels (adjusted for age, sex, and education) and the primary study outcome of longitudinal change in MMSE score. Additional LME models were fit that also included covariate adjustment for CSF Aβ_42_ or P-tau levels. LME models had random intercepts and slopes with an interaction term between time and Aβ_38_ levels and an interaction term between time and Aβ_42_ or P-tau levels for models that also included those biomarkers. Additional analysis of longitudinal cognition was performed in which a demographics-adjusted model including the Aβ_38_/Aβ_40_ ratio was directly compared to a model using the Aβ_42_/Aβ_40_ ratio instead.

Cox regression modeling was used to assess the association between continuous Aβ_38_ levels (adjusted for age, sex, and education) and conversion to AD dementia during longitudinal follow-up. Additional Cox regression models were fit that also included covariate adjustment for either CSF Aβ_42_ or P-tau levels. All participants were right censored (i.e., the last follow-up visit was considered as either the latest visit if the participant was never diagnosed with AD dementia or the visit when diagnosis of AD dementia occurred), and the proportionality of hazards assumption was assessed with Schoenfeld residuals.

All biomarkers and continuous demographic variables were standardized before all model fitting to increase comparability of standardized model coefficients across cohorts. The analysis of longitudinal cognition included all study participants (SCD, MCI, AD), while the analysis of longitudinal risk for AD dementia included only participants who did not already have AD dementia (SCD, MCI).

All code was written in the R programming language (version 4.0.0, R Foundation for Statistical Computing, Vienna, Austria), and all significance tests were 2 sided with α = 0.05 as the significance threshold.

### Research Questions

Our primary research question was whether there was an association between CSF Aβ_38_ and AD-related outcomes evaluated longitudinally in participants with SCD, MCI, or AD who had abnormal AD biomarker signatures. A secondary question was how the association between CSF Aβ_38_ and AD-related outcomes was modulated by further controlling for other Aβ-related biomarkers, P-tau, and *APOE* status.

### Standard Protocol Approvals, Registrations, and Patient Consents

All participants gave written informed consent to participate in the BioFINDER study as approved by the ethics committee of Lund University, Sweden. All participants gave written informed consent to participate in the ADNI study as approved by the ethics committees of all participating sites. All methods were carried out in accordance with the approved guidelines.

### Data Availability

All relevant source data from the present study along with anonymized data from the BioFINDER study will be shared by request from a qualified academic investigator for the sole purpose of replicating procedures and results presented in the article and as long as data transfer is in agreement with European Union legislation on the general data protection regulation and decisions by the Ethical Review Board of Sweden and Region Skåne, which should be regulated in a material transfer agreement. The code used for statistical analyses is available at a public repository.

## Results

### Cohort Characteristics

In the BioFINDER cohort (Table 1), we included 338 biomarker-positive (defined by an elevated CSF Aβ_42_/P-tau ratio, representing 55.8% of 605 eligible participants in the study) participants classified as having SCD (n = 54), MCI (n = 150), or AD (n = 134). The average age was 72.5 ± 6.8 years; 52.1% of participants were female; and average education was 11.0 ± 3.5 years. The average follow-up time was 4.0 ± 1.6 years, with 91.1% of participants having at least a 2-year visit and 65.3% of participants having at least a 4-year visit.

**Table 1:**
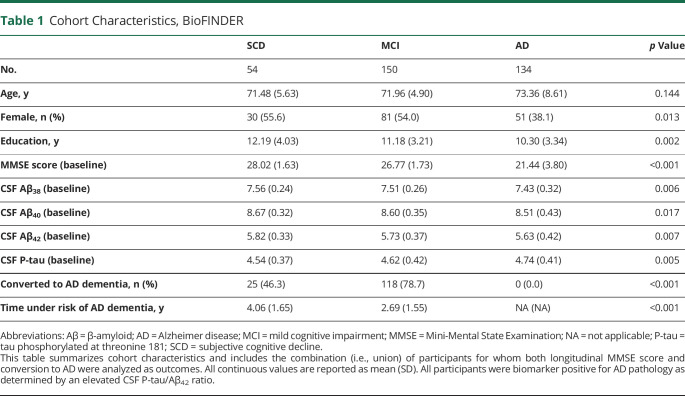
Cohort Characteristics, BioFINDER

In the ADNI cohort (Table 2), we included 318 biomarker-positive participants (47.8% of 665 eligible participants in the study) classified as having SCD (n = 17), MCI (n = 192), or AD (n = 109). The average age was 73.1 ± 7.4 years; 54.7% of participants were female; and average education was 15.9 ± 2.8 years. The average follow-up time was 3.7 ± 2.5 years, with 73.1% of participants having at least a 2-year visit and 49.5% of participants having at least a 4-year visit.

**Table 2:**
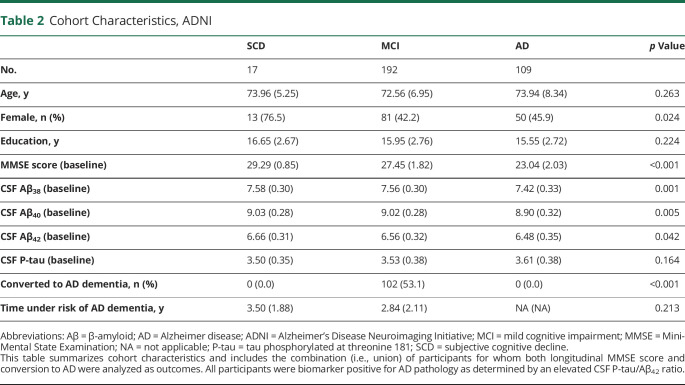
Cohort Characteristics, ADNI

We compared demographic variables between cohorts and found no significant difference in participant age (*p* = 0.28) or in the percentage of male/female participants (*p* = 0.49). However, participants from the ADNI cohort had significantly higher educational attainment than participants from the BioFINDER cohort (difference 4.85 years, *p* < 0.001).

**Figure 1:**
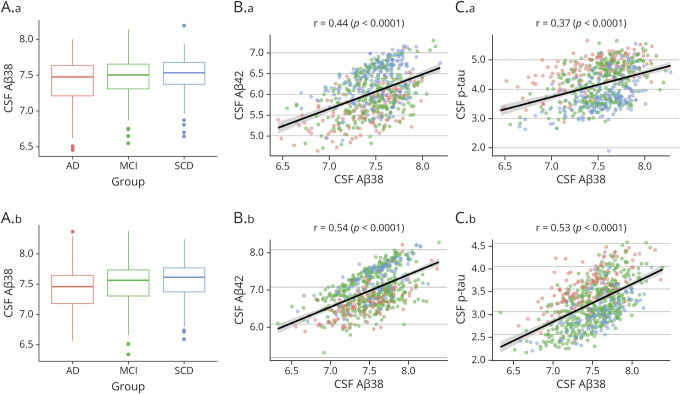
Distribution of CSF Aβ_38_ Levels Across Diagnostic Groups and Cohorts and Their Association With CSF Aβ_42_ and CSF P-tau This figure shows how CSF β-amyloid (Aβ)_38_ levels are distributed across diagnostic groups (A) and how CSF Aβ_38_ levels relate to CSF Aβ_42_ (B) and CSF tau phosphorylated at threonine 181 (P-tau) (C) levels in the BioFINDER (A.a, B.a, C.a) and Alzheimer’s Disease Neuroimaging Initiative (A.b, B.b, C.b) cohorts. Association between biomarkers was tested with the Pearson correlation. Data points in the scatterplots (B and C) are colored according the same scheme in the boxplots (A): red = Alzheimer disease (AD), green = mild cognitive impairment (MCI), and blue = subjective cognitive decline (SCD).

We tested the relationship between biomarkers in both cohorts independently (Figure 1) and found that CSF Aβ_38_ was significantly correlated with Aβ_42_ (*r* = 0.44, *p* < 0.0001 in BioFINDER; *r* = 0.54, *p* < 0.0001 in ADNI) and with P-tau (*r* = 0.37, *p* < 0.0001 in BioFINDER; *r* = 0.53, *p* < 0.0001 in ADNI). Looking across diagnosis in the BioFINDER cohort, we found that the association between CSF Aβ_38_ and CSF Aβ_42_ in the BioFINDER cohort was highest in the AD group (*r* = 0.59) compared to the MCI (*r* = 0.37) and SCD (*r* = 0.41) groups. Meanwhile, in the ADNI cohort, the association between CSF Aβ_38_ and CSF Aβ_42_ was highest in those with SCD (*r* = 0.70) and AD (*r* = 0.66) compared to MCI (*r* = 0.47). While our primary analysis included only biomarker-positive individuals, we found that this association was higher in biomarker-negative individuals (*r* = 0.63). Moreover, CSF Aβ_38_ levels did not significantly differ by *APOE* status in the BioFINDER cohort (*p* = 0.20 for 0 vs 1 ε4 copy, *p* = 0.15 for 0 vs 2 ε4 copies).

### Association With Longitudinal Decline in Cognition

**Figure 2:**
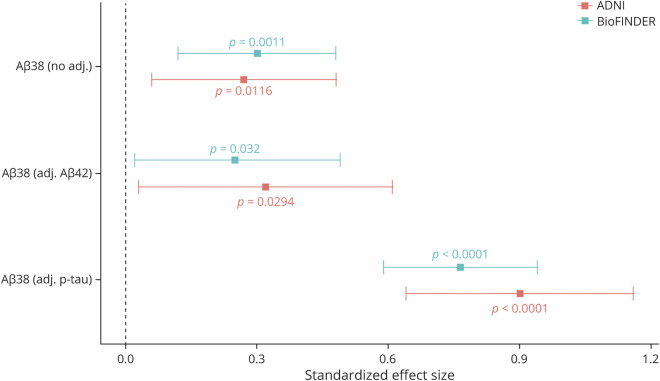
Association Between CSF Aβ_38_ and Longitudinal Cognition Across Cohorts This figure displays results from linear mixed-effects analysis in the BioFINDER and Alzheimer’s Disease Neuroimaging Initiative (ADNI) cohorts to investigate the association between longitudinal Mini-Mental State Examination (MMSE) score and β-amyloid (Aβ)_38_ alone, Aβ_38_ adjusted for Aβ_42_, and Aβ_38_ adjusted for tau phosphorylated at threonine 181 levels. All models were additionally adjusted for age, sex, and education. Coefficients are displayed for the effect of Aβ_38_ both on baseline MMSE score (baseline in the figure) and on change in MMSE over time (slope in the figure).

In the BioFINDER cohort, higher CSF Aβ_38_ levels adjusted for demographics (age, sex, and education) were associated with less decline in MMSE score over time (β = 0.30 points per year per SD of biomarker change, *p* = 0.001). Higher Aβ_38_ levels adjusted for Aβ_42_ levels were also associated with less decline in MMSE score over time (β = 0.25, *p* = 0.03); Aβ_42_ in the same model was not significantly associated with MMSE score change (*p* = 0.57). Higher Aβ_38_ levels with additional adjustment for P-tau were associated with less decline in MMSE score over time (β = 0.76, *p* < 0.0001); P-tau in the same model was also significantly associated with MMSE score change (β = −0.94, *p* < 0.0001). These results are displayed graphically in Figure 2.

In the ADNI cohort, higher Aβ_38_ levels adjusted for demographics (age, sex, and education) were associated with less decline in MMSE score over time (β = 0.27, *p* = 0.01). Higher Aβ_38_ levels with additional adjustment for Aβ_42_ were also associated with less decline in MMSE score over time (β = 0.32, *p* = 0.03); Aβ_42_ in the same model was not significantly associated with MMSE score change (*p* = 0.61). Finally, higher Aβ_38_ levels with additional adjustment for P-tau were associated with less decline in MMSE score over time (β = 0.90, *p* < 0.0001); P-tau in the same model was also significantly associated with MMSE score change (β = −0.95, *p* < 0.0001). These results are displayed graphically in Figure 2.

Using the PACC scale in the ADNI cohort showed that higher Aβ_38_ levels were associated with longitudinal change in PACC adjusted only for covariates (β = 0.37, *p* = 0.016) and adjusted for covariates and CSF P-tau (β = 1.35, *p* < 0.0001) but not when additionally adjusted for CSF Aβ_42_ (β = 0.34, *p* = 0.11).

Moreover, using the Aβ_38_/Aβ_40_ and Aβ_42_/Aβ_40_ ratios directly as predictors showed that neither ratio measure was associated with longitudinal change in MMSE score for the BioFINDER cohort (*p* = 0.34 and *p* = 0.55, respectively). However, there was a significant association for Aβ_38_/Aβ_40_ (β = 0.30, *p* = 0.0056), but not Aβ_42_/Aβ_40_ (*p* = 0.39), for the same outcome in the ADNI cohort. These results were qualitatively similar when the composite PACC score was used as a cognitive outcome.

Last, we tested the association of CSF Aβ_38_ with the outcomes of interest while controlling for additional variables of interest. When controlling directly for diagnostic status with longitudinal cognition as outcome, CSF Aβ_38_ levels adjusted for age, sex, education, and diagnostic status were significantly associated with MMSE score (β = 0.30, *p* = 0.001) but not PACC score (β = −0.06, *p* = 0.31) in the BioFINDER cohort and significantly associated with both MMSE score (β = 0.22, *p* = 0.036) and PACC score (β = 0.30, *p* = 0.045) in the ADNI cohort. It is important to note that the overall model *R*^2^ increased greatly when we included diagnostic status as outcome, whereas the standardized regression coefficient for Aβ_38_ was generally smaller. Moreover, there was no significant change in association between Aβ_38_ levels and longitudinal cognition for any model when controlling directly for number of *APOE* ε4 copies.

### Association With Risk of AD Dementia

**Figure 3:**
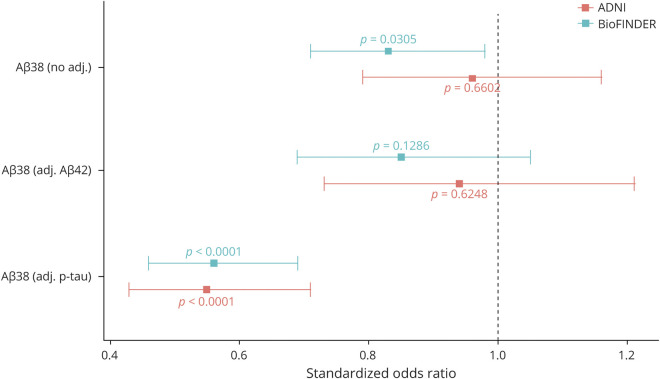
Association Between CSF Aβ_38_ and Clinical Conversion Across Cohorts This figure displays results from Cox regression analysis in the BioFINDER and Alzheimer’s Disease Neuroimaging Initiative (ADNI) cohorts to investigate the association between risk of developing Alzheimer disease (AD) dementia and β-amyloid (Aβ)_38_ alone, Aβ_38_ adjusted for Aβ_42_, and Aβ_38_ adjusted for tau phosphorylated at threonine 181 levels. All models were additionally adjusted for age, sex, and education. Coefficients represent the change in odds of converting to AD dementia for each SD increase in Aβ_38_ levels.

In the BioFINDER cohort, higher Aβ_38_ levels adjusted for demographics were associated with lower risk of AD dementia (hazard ratio [HR] 0.83 higher odds per SD of biomarker change [95% confidence interval 0.71–0.98], *p* = 0.03), while higher Aβ_38_ levels additionally adjusted for Aβ_42_ trended toward an association with lower risk of conversion (HR 0.85 [0.69–1.05], *p* = 0.12), and higher Aβ_38_ levels additionally adjusted for P-tau were strongly associated with lower risk of conversion (HR 0.56 [0.46–0.69], *p* < 0.0001). These results are displayed graphically in Figure 3.

In the ADNI cohort, there was not a significant association with conversion to AD dementia when Aβ_38_ levels were adjusted only for demographics (HR 0.96 [0.79–1.16], *p* = 0.66), and there was not a significant association when Aβ_38_ was adjusted for demographics and Aβ_42_ (HR 0.94 [0.73–1.21], *p* = 0.62). Still, higher Aβ_38_ levels adjusted for demographics and P-tau were strongly associated with lower risk of conversion to AD dementia (HR 0.55 [0.43–0.71], *p* < 0.0001). These results are displayed graphically in Figure 3.

### Analysis of Other Aβ Biomarkers

We performed the same analyses and in the same groups but using CSF Aβ_40_ as the variable of interest instead of CSF Aβ_38_. In BioFINDER, higher CSF Aβ_40_ levels adjusted only for demographics were associated with less decline in MMSE score (β = 0.30 points per year per SD of biomarker change, *p* = 0.01; not significantly different from the effect size of Aβ_38_, *p* = 0.48). Aβ_40_ levels were also associated with less decline in MMSE score when adjusted additionally for CSF Aβ_42_ (β = 0.27, *p* = 0.04) and CSF P-tau (β = 0.72, *p* < 0.0001). In ADNI, higher Aβ_40_ levels adjusted for only demographics were not associated with higher change in MMSE score (β = 0.21, *p* = 0.051) or when also adjusted for CSF Aβ_42_ (β = −0.09, *p* = 0.15) but were significant when adjusted for CSF P-tau (β = 0.84, *p* < 0.0001). The standardized effect sizes for Aβ_40_ in the ADNI cohort were smaller in magnitude for all models than those that instead included Aβ_38_.

With regard to conversion to AD dementia in the BioFINDER cohort, CSF Aβ_40_ levels adjusted only for demographics were weakly associated with conversion to AD dementia (HR 0.84 lower odds per SD of biomarker change, *p* = 0.048). As with Aβ_38_, higher Aβ_40_ levels were not associated with conversion to AD dementia when adjusted further for Aβ_42_ (HR 0.86, *p* = 0.21) but did have a significant association when adjusted further for P-tau (HR 0.61, *p* < 0.0001). The standardized effect size for Aβ_40_ was smaller for all modes in the BioFINDER cohort compared to the same models with Aβ_38_. In ADNI, CSF Aβ_40_ levels were not associated with conversion to AD dementia when adjusted only for demographics (HR 1.03, *p* = 0.78) or additionally for Aβ_42_ (HR = 1.08, *p* = 0.61) but were significantly associated when adjusted further for P-tau (HR 0.60, *p* = 0.0003). The standardized effect size for significant Aβ_40_ models in the ADNI cohort was again smaller than the corresponding models that included Aβ_38_ instead.

A similar analysis of CSF sAPP levels (available only in BioFINDER) showed no association with change in MMSE score (β = −0.01, *p* = 0.94) or conversion to AD dementia (HR = 0.94, *p* = 0.65).

## Discussion

There is a great need in the AD research field to explain why overwhelming evidence points to amyloid being the key driver of AD pathogenesis while antiamyloid therapies have had, until the present day, only rather modest effect on disease progression in late-stage clinical trials. One proposed explanation for recent trial results has been to question inherent factors of the trials themselves, e.g., inclusion criteria, choice of endpoint, too late treatment initiation, or statistical power.^25^ However, more recent AD trials have included stringent biomarker inclusion criteria, sophisticated composite clinical endpoints, and large numbers of participants.^7,8,26^

Assuming then that these trial failures are due to biological factors, another response has been to instead question the entire amyloid cascade hypothesis by suggesting that amyloid accumulation may be an indirect effect rather than primary cause of AD.^27^ However, the existence of early-onset familial AD caused by mutations in the *APP*, *PSEN1*, and *PSEN2* genes—all part of the Aβ processing machinery—suggests that the true explanation for the lack of immediate success of antiamyloid therapies should still retain the integrity of the amyloid cascade hypothesis.^28^ One possible mechanism could be that there is a more complex interaction between different Aβ peptides than previously appreciated, which would explain why targeting of Aβ_42_ alone may not be sufficient to halt the disease progression. Recent evidence suggests that lower levels of shorter Aβ peptide levels or a lower ratio of shorter to longer Aβ peptides could be an important factor in Aβ toxicity.^11,15,16^

The results of our current study support this hypothesis from a clinical perspective in that we demonstrated that higher CSF Aβ_38_ levels are associated with less cognitive decline and lower risk of developing AD dementia in individuals who are biomarker positive (Table 3 summarizes the evidence). The adjustment of our statistical models for core AD biomarkers (Aβ_42_, P-tau) despite using them as inclusion criteria for the present analysis reflects our attempt to handle the idea that binary cutoffs must necessarily be used in patient workflows, but for prognostic modeling, it is best to use continuous biomarker values. Our finding that higher CSF Aβ_38_ levels are protective even in the presence of significant AD pathology in the brain (we included only biomarker-positive individuals) should motivate further studies to understand the molecular underpinning of a potential protective mechanism from Aβ_38_ and possibly even Aβ_40_. For instance, it is unclear whether Aβ_38_ levels modulate the development of tau pathology in individuals who already reached thresholds for Aβ positivity pathology. It is interesting to note that we also found that the Aβ_38_/Aβ_40_ ratio was a stronger predictor of longitudinal cognitive decline than Aβ_42_/Aβ_40_ in individuals with AD pathology.

**Table 3:**
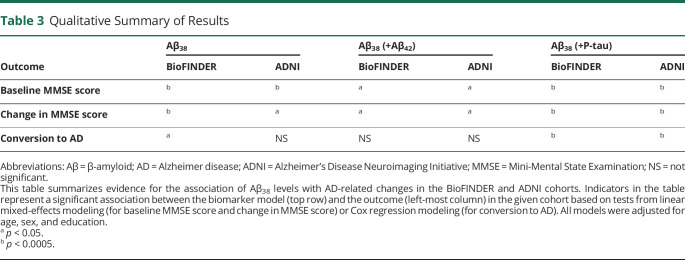
Qualitative Summary of Results

The validation of our findings in 2 independent cohorts with differing demographic profiles—ADNI participants have high educational attainment on average and are primarily typical amnestic AD cases while the BioFINDER cohort is more heterogeneous in demographic and diagnostic makeup—adds validity to our results. Still, the validation is strengthened by the finding that standardized effect sizes of Aβ_38_ were similar across cohorts (e.g., β = 0.30 for Aβ_38_ in BioFINDER and β = 0.27 for Aβ_38_ in ADNI, for longitudinal MMSE score as outcome). It is important to note that the effect sizes for Aβ_38_ in the statistical models were generally stronger than those for Aβ_40_ or sAPP, indicating a specific effect of Aβ_38_ rather than simply an effect of total Aβ production or APP cleavage. Taken together, then, it is unlikely that our findings could be due to systematic changes related to CSF collection, volume, or measurement. Our results were also largely replicated across 2 different cognitive scales in the ADNI cohort, indicating that choice of cognitive measure should not affect results significantly.

These findings are of particular importance due to the renewed interest in GSMs, a class of drugs that reduce Aβ_42_ production while maintaining total Aβ production by blocking cleavage of APP at specific γ-secretase cleavage sites.^29,30^ Compared to previously tested γ-secretase inhibitors, which had untenable off-target effects in past clinical trials, GSMs do not alter total Aβ production and thus do not compromise the broader biological role of γ-secretase.^30,31^ Previous studies of GSMs have shown that the Aβ_38_ peptide does not exhibit any toxicity in vivo (nor does it accumulate into plaques after overexpression in mice) and can even protect against Aβ_42_-associated dysfunction.^15^ However, while the Aβ_42_/Aβ_40_ ratio has been widely implicated in both clinical studies of AD and animal studies of GSMs (primarily as a proxy for brain Aβ buildup), few clinical studies before ours have investigated Aβ_38_ levels.^32-34^

Unfortunately, no GSM compound has yet been brought to a phase III clinical trial. However, a review of the literature reveals that hindrances of GSMs in early-phase trials relate largely to poor penetrance into the brain or economic concern.^35,36^ Nonetheless, work on GSM compounds within the AD field remains active with regard to preclinical animal studies,^37^ in vitro studies demonstrating significant effects on amyloid processing and accumulation in relevant disease models,^38^ and computational studies investigating potential modulator binding sites or mechanisms of action leading to identification of clinical candidates.^39,40^ Due to the accumulating evidence that GSM compounds affect amyloid processing, it is likely that these drugs will be targeted toward individuals with familial AD. Therefore, it is important to closely follow results from ongoing studies in such populations such as the Dominantly Inherited Alzheimer Network Trials Unit (DIAN-TU) prevention trial.^41^

Our results suggest that further investigations should be undertaken to understand whether increasing the relative levels of shorter Aβ peptides such as Aβ_38_ is in fact an effective strategy to treat AD.^42,43^ We must note that we did not test whether CSF Aβ_38_ has added clinical value over well-established biomarkers of amyloid, tau, and neurodegeneration. Instead, we provided clinical evidence here that higher Aβ_38_ levels are in fact associated with lower risk of AD-related changes, which may support the use of GSMs as an approach to altering AD progression. Still, our understanding of the interaction between the different Aβ peptides is still lacking. In addition, we restricted our analysis to AD biomarker–positive individuals for multiple reasons. First, rates of cognitive decline and AD dementia are low among AD biomarker–negative individuals.^44^ Second, biomarker-positive individuals are the target for nearly all disease-altering therapies in AD; therefore, we aimed to understand whether Aβ_38_ levels modulate cognitive decline in this highly relevant population.

The present study was focused only on individuals with abnormal biomarker pathology because those without abnormal biomarker pathology are highly likely to remain stable within the timescales of our study (2–6 years) from both a cognitive and a clinical standpoint. Change in shorter amyloid peptides in healthy, elderly individuals who may be relevant for longer-term preventive AD trials is thus outside the scope of the current study and is a subject for further investigation.

## Publication History

This manuscript was prepublished in medrxiv.org/content/10.1101/2021.01.31.21250702v1. Received by *Neurology* May 3, 2021. Accepted in final form December 8, 2021.

## Glossary

Aβ: β-amyloid
AD: Alzheimer disease
ADNI: Alzheimer's Disease Neuroimaging Initiative
DIAN-TU: Dominantly Inherited Alzheimer Network Trials Unit
GSM: γ-secretase modulator
HR: hazard ratio
LME: linear mixed-effects
MCI: mild cognitive impairment
MMSE: Mini-Mental State Examination
P-tau: tau phosphorylated at threonine 181
PACC: Preclinical Alzheimer's Cognitive Composite
sAPP: amyloid precursor protein
SCD: subjective cognitive decline
